# The VEGF‐Mediated Cytoprotective Ability of MIF‐Licensed Mesenchymal Stromal Cells in House Dust Mite‐Induced Epithelial Damage

**DOI:** 10.1002/eji.202451205

**Published:** 2024-11-06

**Authors:** Hazel Dunbar, Ian J. Hawthorne, Courteney Tunstead, Molly Dunlop, Evelina Volkova, Daniel J. Weiss, Claudia C. dos Santos, Michelle E. Armstrong, Seamas C. Donnelly, Karen English

**Affiliations:** ^1^ Kathleen Lonsdale Institute for Human Health Research Maynooth University Maynooth, Co. Kildare Ireland; ^2^ Department of Biology Maynooth University Maynooth, Co. Kildare Ireland; ^3^ Department of Medicine, 226 Health Sciences Research Facility, Larner College of Medicine University of Vermont Burlington Vermont USA; ^4^ The Keenan Research Centre for Biomedical Science of St. Michael's Hospital Toronto Ontario Canada; ^5^ Institute of Medical Sciences and Interdepartmental Division of Critical Care Faculty of Medicine University of Toronto Toronto Ontario Canada; ^6^ Department of Medicine Trinity College Dublin and Tallaght Hospital Dublin Ireland

**Keywords:** airway epithelial, house dust mite, macrophage migration inhibitory factor, mesenchymal stromal cells, vascular endothelial growth factor

## Abstract

Enhancing mesenchymal stromal cell (MSC) therapeutic efficacy through licensing with proinflammatory cytokines is now well established. We have previously shown that macrophage migration inhibitory factor (MIF)‐licensed MSCs exerted significantly enhanced therapeutic efficacy in reducing inflammation in house dust mite (HDM)‐driven allergic asthma. Soluble mediators released into the MSC secretome boast cytoprotective properties equal to those associated with the cell itself. In asthma, epithelial barrier damage caused by the inhalation of allergens like HDM drives goblet cell hyperplasia. Vascular endothelial growth factor (VEGF) plays a pivotal role in the repair and maintenance of airway epithelial integrity. Human bone marrow‐derived MSCs expressed the MIF receptors CD74, CXCR2, and CXCR4. Endogenous MIF from high MIF expressing CATT_7_ bone marrow‐derived macrophages increased MSC production of VEGF through the MIF CXCR4 chemokine receptor, where preincubation with CXCR4 inhibitor mitigated this effect. CATT_7_‐MIF licensed MSC conditioned media containing increased levels of VEGF significantly enhanced bronchial epithelial wound healing via migration and proliferation in vitro. Blocking VEGFR2 or the use of mitomycin C abrogated this effect. Furthermore, CATT_7_‐MIF MSC CM significantly decreased goblet cell hyperplasia after the HDM challenge in vivo. This was confirmed to be VEGF‐dependent, as the use of anti‐human VEGF neutralising antibody abrogated this effect. Overall, this study highlights that MIF‐licenced MSCs show enhanced production of VEGF, which has the capacity to repair the lung epithelium.

## Graphical abstract

Licensing of MSCs with MIF requires CXCR4 receptor binding. Enhanced VEGF production by MIF‐licensed MSCs promotes wound healing in vitro and can be blocked using the VEGFR2 inhibitor SU‐5416. MIF‐licensed MSC conditioned medium significantly reduced goblet cell hyperplasia in house dust mite‐driven allergic airway inflammation in vivo.

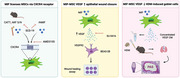

## 1 Introduction

Mesenchymal stromal cells (MSCs) are renowned for their cytoprotective abilities elicited through secreted factors including miRNA [1], mitochondrial DNA [2, 3, 4], lipids (prostaglandin E2), extracellular vesicles (EV) [5, 6, 7], metabolites (kynurenine) and cytokines (TNF and IL‐6) [8, 9, 10]. Furthermore, newly emerging data outlines the importance of MSC‐derived apoptotic bodies in their therapeutic efficacy, making the secretome their main mechanism of action [11, 12, 13, 14, 15]. MSCs are known to elicit their anti‐apoptotic and pro‐regenerative effects through the production of vascular endothelial growth factor (VEGF) [16, 17, 18] and have been shown to enhance B cell survival in a VEGF‐dependent manner [19].

In preclinical models of lung disease, conditioned media from MSCs (MSC CM) has proven to be as potent as the cellular counterpart [5, 20, 21, 22, 23]. After lipopolysaccharide (LPS)‐induced injury, bone marrow‐derived MSC CM mitigated neutrophil influx and alternatively activated wound healing associated M2 alveolar macrophages, dampening lung injury in an IGF‐1 dependent manner [24]. Similarly, murine MSC CM rescued lung fibroblasts from a cigarette‐smoked‐induced lung injury, illustrating the positive role of MSC‐secreted factors in facilitating epithelial regeneration [22, 23, 25]. More recent studies also follow this narrative, demonstrating the potent effects of MSC CM in preclinical lung disease [26, 27, 28, 29].

Extensive literature illustrates the benefits of licensing MSCs prior to administration [30, 31, 32, 33, 34, 35, 36, 37], which can be carried out by modifying the environment in which these cells grow through exogenous stimulation, genetic manipulation, or even the addition of chemical agents. The asthmatic lung can act as a suitable environment for the activation of MSCs in vivo, as it contains a multitude of pro‐inflammatory cytokines including macrophage migration inhibitory factor (MIF) [34]. MIF has not only been found at elevated levels in the bronchoalveolar lavage fluid of asthma patients [38] but its level of expression has been linked to disease severity [39]. Low expression of MIF is associated with a low number of repeats of the tetranucleotide repeat polymorphism ‘CATT’ located within the promoter region of the MIF gene [40]. Using novel humanised MIF mice expressing the 7‐repeat allele termed CATT_7_, we have shown that high expression of human MIF drives airway inflammation following the house dust mite (HDM) challenge [41]. Our group has also demonstrated the ability of CATT_7_‐MIF licensing to enhance MSCs immunomodulatory effects in vivo [42].

Epithelial cells lining the airways play a key role in defence mechanisms against external pathogens and thus, disease [43]. In asthmatics, repetitive mechanical exacerbations due to inhaled agents or non‐specific stimuli can result in physical or biological injury of the airways and/or abnormal cycles of wound healing [44, 45, 46], where epithelial cell apoptosis and damage can drive further airway remodelling [47, 48]. MSCs have illustrated efficacy in resolving damage inflicted by these repeated exacerbations by repairing endothelial barrier integrity [49] and increasing wound healing [50].

This study sets out to investigate the capacity for MIF‐licensing to enhance the in vitro and in vivo cytoprotective functions of the MSC secretome and to identify the mechanisms involved using a clinically relevant HDM‐induced allergic airway model.

## 2 Materials and Methods

### 2.1 Ethical Approval and HPRA Compliance

All procedures involving the use of animals were carried out by licensed personnel. Ethical approval for all work was granted by the ethics committee of Maynooth University (BRESC‐2018‐13). Project authorisation was received from the HPRA (AE19124/P022), whereby animal experiments were carried out in accordance with the Animal Research: Reporting of In Vivo Experiments (ARRIVE) criteria.

### 2.2 Cell Culture

Human bone marrow‐derived mesenchymal stromal cells (hBM‐MSCs) (RoosterBio Frederick, MD, USA) were expanded for two passages according to the manufacturer's instructions. Following this, MSCs were cultured and maintained in DMEM low glucose (Sigma‐Aldrich, Arklow, Wicklow, Ireland) supplemented with 10% (v/v) fetal bovine serum (BioSera, Cholet, France) and 1% (v/v) penicillin/streptomycin (Sigma‐Aldrich, Arklow, Wicklow, Ireland). Human alveolar epithelial cells (A549) and human normal bronchial epithelial cells (BEAS‐2B) were cultured in complete low glucose DMEM (Sigma‐Aldrich, Arklow, Wicklow, Ireland). All cells were incubated at 37°C/5% CO_2_/20% O_2_.

### 2.3 Generation of L929 Conditioned Media (M‐CSF)

L929 cells were thawed, seeded in complete RPMI‐1640 medium GlutaMAX (Gibco, Paisley, UK), and incubated at 37°C/5% CO_2_/20% O_2_ for 7 days. The supernatant was collected, centrifuged, and filtered (0.2 µm), and conditioned media containing M‐CSF was aliquoted and stored at −80°C. L929 conditioned media will be referred to as M‐CSF throughout the text.

### 2.4 Surface Staining of MIF Receptors

MSCs were seeded at 1 × 10^5^ cells per well in six‐well plates. MSCs were stimulated with rhMIF (1, 10, or 100 ng/mL), or endogenous MIF (CATT_7_ CM) for 24 h. Cells were stained with CD74 (PE, BD Pharmingen, Berkshire, UK), CXCR2 (CD182) (PerCP‐eFluor 710, eBioscience, San Diego, CA, USA), or CXCR4 (CD184) (APC, eBioscience, San Diego, CA, USA) for 45 min. Cells were then washed in flow cytometry staining buffer and acquired using the Attune Nxt flow cytometer.

### 2.5 Animal Strains

A C57BL/6 mouse strain expressing the human high‐expression CATT_7_ MIF allele (M*IF*CATT_7_ [(C57BL/6NTac‐Miftm3884.1(MIF)Tac‐Tg(CAG‐Flpe)2Arte] mice) was created using vector‐based recombinant replacement of murine MIF by Taconic Biosciences (Rensselaer, NY, USA). The entire mouse MIF promoter has been deleted and replaced by inserting the human MIF promoter region. Validation of human but not murine MIF mRNA expression was verified by qPCR, and −794 CATT‐length dependent stimulated MIF production was confirmed in vivo [51]. Littermate wildtype (WT) and MIF−/− (MIF knockout) mice (R. Bucala, Yale School of Medicine, Yale University, New Haven, CT, USA) were used as controls. All mice were housed according to the HPRA SAP (Ireland) guidelines.

### 2.6 Model of HDM‐Induced Allergic Airway Inflammation

CATT_7_ transgenic humanised MIF mice were intranasally (I.N.) challenged with 25 µg of *Dermatophagoides pteronyssinus* (endotoxin content of 9937.5 EU/vial) (Greer Laboratories Inc, Lenoir, NC, USA) on days 0, 2, 4, 7, 9, 11, 14, 16, and 18.

### 2.7 Histological Analysis

On day 21 of the HDM model, lungs were harvested. Tissue was fixed in 10% (v/v) neutral buffered formalin (Sigma‐Aldrich) for 24 h, processed, and embedded in paraffin wax (Shandon Pathcentre, Runcorn, UK). For periodic acid Schiff (PAS) (Abcam, Cambridge, UK) staining, tissue sections (5 µm) were stained, air dried, and a coverslip mounted with DPX mounting media (Sigma‐Aldrich, Wicklow, Ireland). 20× images were taken using an Olympus BX51 light microscope. Images were scored by counting the number of PAS‐positive mucin‐producing goblet cells, relative to the diameter of the airway in a blinded manner.

### 2.8 Generation of CATT7 MIF Conditioned Media

Mice were humanely euthanised using the cervical dislocation technique on day 18, 4 h after the last HDM challenge. Bone marrow was isolated from the femur and tibia and centrifuged at 300*g* for 5 min and red blood cells were lysed (eBioscience, San Diego, CA, USA). Cells were seeded in two T175 flasks per mouse. Cells were grown for 72 h in complete RPMI‐1640 medium GlutaMAX (Gibco) supplemented with 20% L929 conditioned media. Supernatants were collected, centrifuged, and filter sterilised to remove cell debris. Aliquots were stored at −20°C. Aliquots were not freeze‐thawed. To account for the variability of human MIF levels between CATT_7_ mice and to verify that WT mice did not produce human MIF, supernatants were measured by human MIF ELISA (R&D) [41].

### 2.9 Generation of MSC Conditioned Media

hBM‐MSCs were cultured as described. Documented concentrations of rhMIF (1, 100, or 400 ng/mL) [52, 53] or conditioned media generated by bone marrow‐derived macrophages (BMDMs) from CATT_7_, WT, or MIF−/− mice were added with fresh cDMEM at a 1:1 ratio for 24 h. Cells were washed with warm PBS and replaced with serum‐free media. After 48 h, supernatants were collected and centrifuged to remove cell debris. Aliquots were stored at −20°C.

### 2.10 Generation of MIF Inhibited SCD‐19 Conditioned Media

SCD‐19 (3‐(2‐methylphenyl)‐1H‐isochromen‐1‐one) (Specs.net, the Netherlands) was reconstituted in 70% ethanol and diluted in PBS to a working concentration of 100 µM. SCD‐19 was added to BMDM‐derived CATT_7_ and WT supernatant for 1 h in a shaking incubator at 37°C before the supernatants were added at a 1:1 ratio into flasks containing human BM‐MSCs.

### 2.11 Wound Healing Assay

The underside of a six‐well plate (Sarstedt, Nümbrecht, Germany) was scratched with three horizontal lines using a scalpel and a ruler to allow for accurate analysis. A549 or BEAS‐2B cells were seeded out at a density of 1 × 105/mL/well. When cells are 60–80% confluent, a single perpendicular vertical scratch was made with a sterile p200 tip. Wells were washed with warm PBS to remove cell debris. cDMEM and MIF‐MSC conditioned media were added in a 1:1 ratio. On day 0, baseline measurements (100% open) were taken using Optika imaging software and a Nikon imaging microscope. Plates were incubated at 37°C/5% CO2/20% O2 for 48 h, or until one scratch had sufficiently closed. Cells were fixed with 10% neutral buffered formalin for 8 min, air dried, and stained with crystal violet (Sigma) for 4 min. ImageJ software was used to measure the percentage of wound closure of each image (Figure S1), relative to the 100% baseline measurements taken on day 0 (Table S1).

### 2.12 Use of VEGFR2 Inhibitor SU‐5416

To investigate if VEGF was facilitating MSCs’ ability to enhance wound closure in A549 and BEAS‐2B epithelial cells, a VEGFR2 inhibitor SU‐5416 (Tocris) was used. 10 µM of SU‐5416 or a DMSO vehicle control was added to A549 or BEAS‐2B cells for 4 h before the scratch was created and conditioned media was added.

### 2.13 Use of Cell Cycle Inhibitor Mitomycin C

BEAS‐2B cells were exposed to 10 µg/mL Mitomycin C (MMC), for 2 h, before being washed off with PBS. Cells were then scratched and a conditioned medium was added.

### 2.14 Preparation of CATT_7_ MSC CM for In Vivo Administration

MSC CM and CATT_7_ CM were generated as previously described. To concentrate the levels of human VEGF present, Amicon Ultra‐0.5 Centrifugal Filter Units (Sigma‐Aldrich) with a molecular weight cut‐off of 50 kDa were used as per the manufacturer's instructions. An anti‐human VEGF neutralising antibody (Bevacizumab Biosimilar) or human IgG1 isotype control was added to the concentrated conditioned media. 30 µL of conditioned media was administered intranasally on day 14 of the HDM model.

### 2.15 VEGF Elisa

MIF‐licensed MSC supernatants were collected and centrifuged at 300*g* for 5 min to remove debris, before being stored at −20°C. ELISAs were carried out according to the manufacturer's instructions (R&D Systems).

### 2.16 Gene Expression Analysis

At the 48 h timepoint, the cells were also harvested for gene expression studies of genes that indicate proliferation: *pcna* and *mib1/ki67* (detailed in Table S2). Total RNA was extracted using TRIzol (Ambion Life Sciences) according to the manufacturer's instructions. cDNA synthesis was performed using the manufacturer's instructions (Quantobio cDNA synthesis kit). Real‐time‐polymerase chain reaction (RT‐PCR) was carried out using PerfeCta SYBR Green FastMix (Quantbio). Expression was quantified in relation to the housekeeper gene HPRT using the ΔCT method. The fold change in the relative gene expression was determined by calculating the 2^−ΔΔCT^ values.

### 2.17 Statistical Analysis

Mice were randomised. Observers assessing end‐points were blinded to group assignments. Data for individual animals and independent experiments are presented as individual symbols. All data are presented as mean ± SEM. Results of two or more groups were compared by one‐way or two‐way analysis of variance (ANOVA) followed by the post hoc Tukey's multiple comparison test. GraphPad Prism (GraphPad Software Inc., San Diego, CA, USA) was used for all statistical analyses.

## 3 Results

### 3.1 Human BM‐MSCs Constitutively Express Canonical and Non‐Canonical MIF Receptors

**FIGURE 1:**
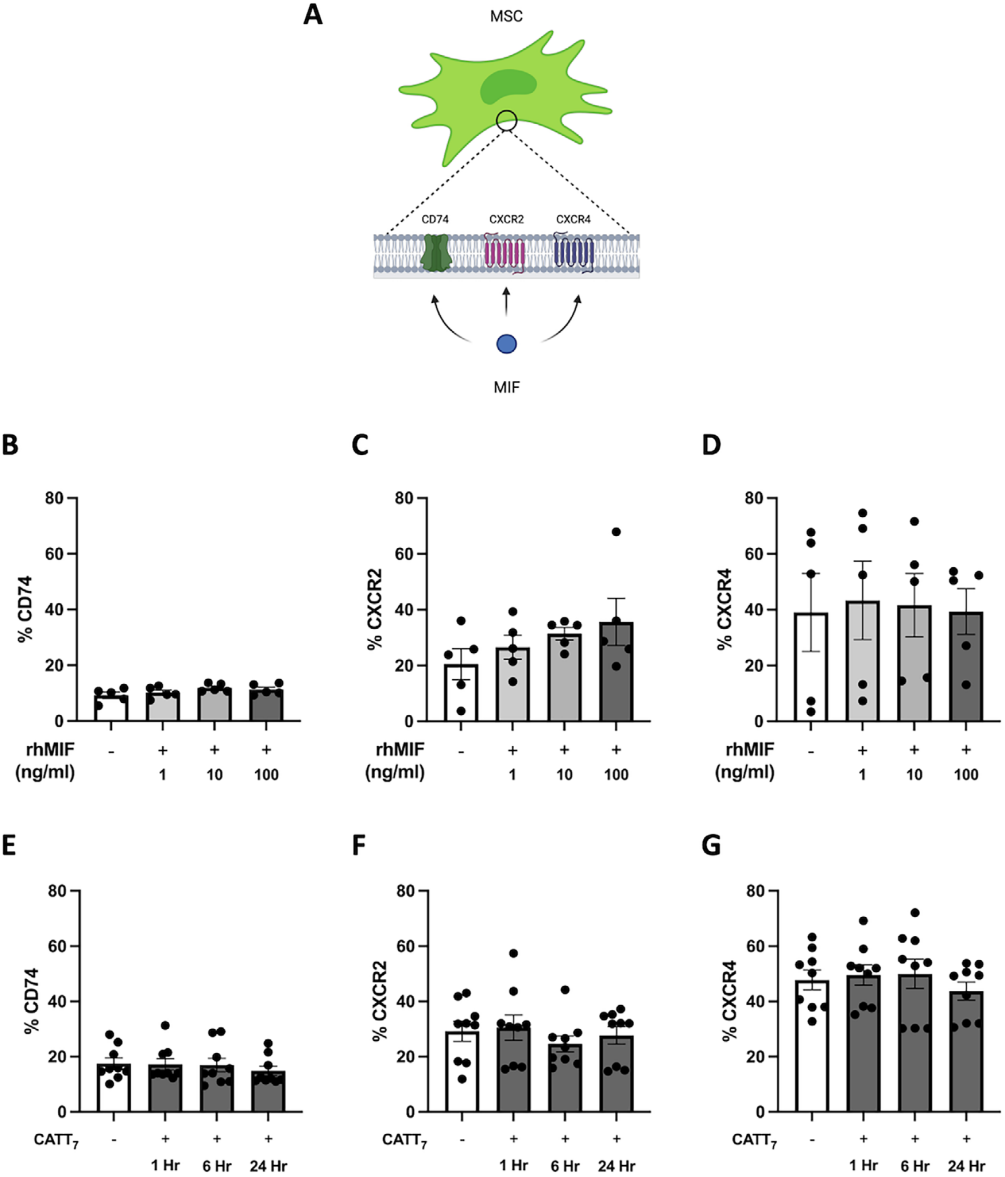
Human BM‐MSCs constitutively express canonical and non‐canonical MIF receptors. A, Human BM‐MSCs expressing MIF receptors CD74, CXCR2, and CXCR4 on their cell surface. MSCs were treated with different concentrations of rhMIF (1, 10, and 100 ng/mL) for 6 h, and percentage of (B) CD74, (C) CXCR2, (D) CXCR4 expression were measured by flow cytometry. Additionally, MSCs were treated with endogenous hMIF generated from BMDMs from CATT_7_ HDM challenged mice (CATT_7_ CM) where the percentage of (E) CD74, (F) CXCR2, (G) CXCR4 expression was assessed after endogenous hMIF stimulation at 6, 12, and 24 h timepoints. Data are presented as mean ± SEM and represent three independent experiments; no statistically significant differences were found by one‐way ANOVA.

To explore the interaction between MIF and MSCs, we sought to determine whether MSCs express the classical (CD74) and non‐classical MIF receptors (CXCR2 and CXCR4) by flow cytometric analysis (Figure 1A). MSCs constitutively expressed MIF receptors CD74, CXCR2, and CXCR4, with CXCR4 most highly expressed (Figure 1). Interestingly, there was large inter‐donor variability in CXCR4 expression and this aligns with other studies [54].

**FIGURE 2:**
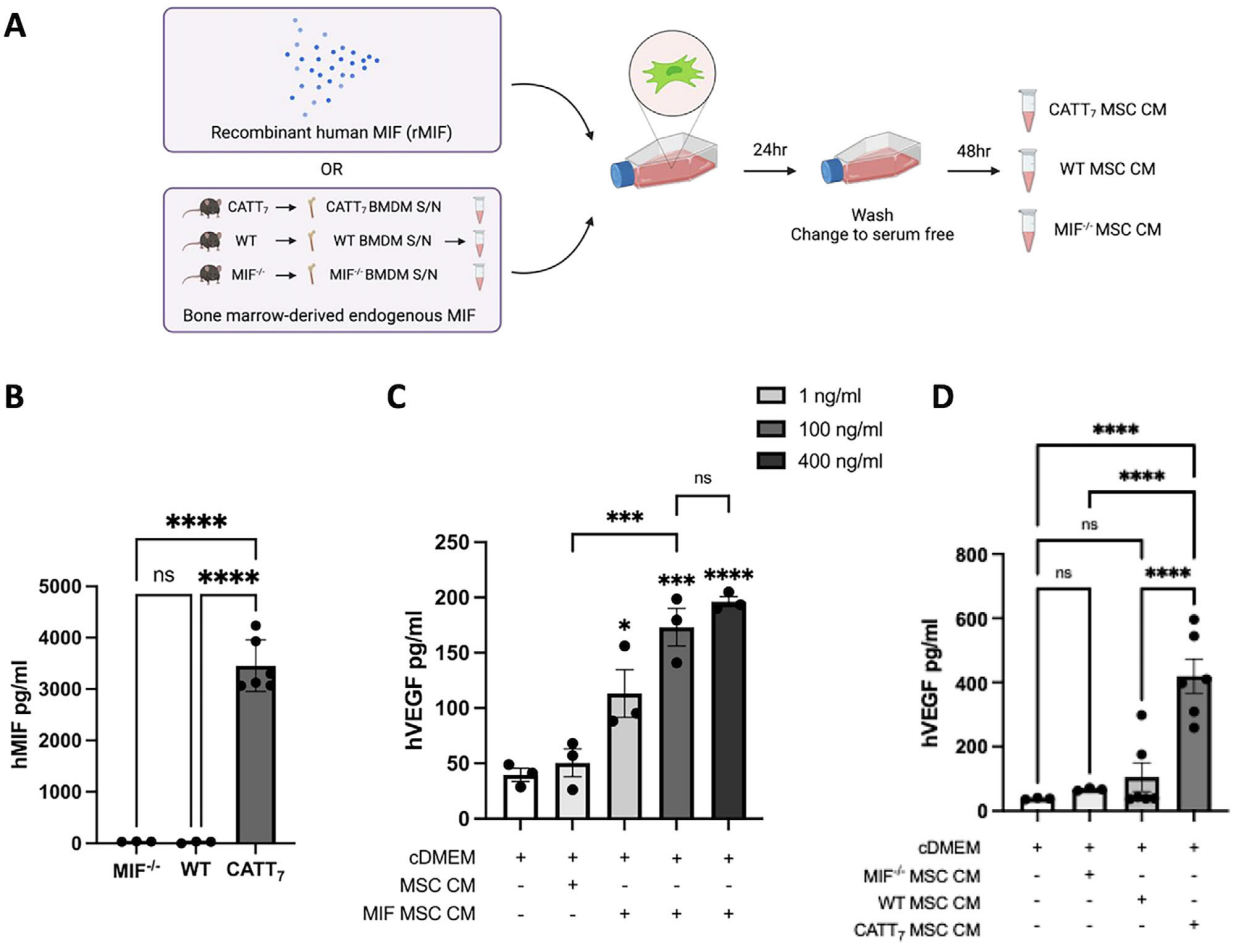
Recombinant or endogenous MIF stimulates MSC secretion of VEGF. A, Schematic depicting the generation of MIF MSC conditioned media using recombinant human MIF or endogenous human MIF generated from BMDMs of CATT_7_ transgenic mice that were challenged with HDM for 3 weeks. HDM‐challenged WT and MIF knockout (MIF^−/−^) mice that do not express human MIF were used as controls. B, Levels of hMIF detected in the BMDMs of MIF^−/−^, WT, and CATT_7_ mice challenged with 25 µg of HDM for 3 weeks. (*n* = 3–6 mice). (C) Human VEGF protein levels in MSC conditioned media supernatants, measured by ELISA, after licensing with different concentrations of recombinant human MIF (1, 100, and 400 ng/mL). (D) Human VEGF protein levels in MIF MSC conditioned media supernatants, using bone marrow‐derived supernatants from high MIF expressing CATT_7_ mice, MIF^−/‐^ mice, or WT mice (*n* = 3–6). Data are presented as mean ± SEM and represent three independent experiments. Statistical significance was determined by one‐way ANOVA; ns = non‐significant; *p < 0.05, ***p < 0.001, ****p < 0.0001.

Next, we investigated if MIF binding influences receptor expression. Recombinant human MIF (rhMIF) did not affect the MSC expression levels of MIF receptors CD74 (Figure 1B), CXCR2 (Figure 1C), or CXCR4 (Figure 1D). We also utilised endogenous MIF from our humanised CATT_7_ MIF mice as an alternative method of licensing MSCs (Figure 2A), a process which we have previously shown significantly enhances MSC therapeutic efficacy [42]. As expected, CATT_7_ mice that express the high MIF expression allele produce significantly higher levels of human MIF compared with WT controls (Figure 2B). Similar to the observations with rhMIF (Figure 1B–D), exposure to high levels of endogenous MIF from CATT_7_ CM across several timepoints (1, 6, and 24 h) had no effect on the percentage of CD74 (Figure 1E), CXCR2 (Figure 1F), and CXCR4 (Figure 1G) expression on the cell surface of MSCs. Thus, these data indicate that MSCs have the ability to interact with human MIF, through both the classical CD74 receptor, but also non‐classical chemokine CXCR2 and CXCR4 receptors.

### 3.2 Recombinant and Endogenous MIF Stimulates MSC Secretion of VEGF

Activation of MSCs boosts their cytoprotective effects through the production of soluble mediators [55, 56]. MSCs were licensed with different concentrations of rhMIF (1, 100, or 400 ng/mL) or with BMDM supernatants from HDM‐challenged human MIF‐expressing CATT_7_ mice, MIF^−/−^ and WT mice (Figure 2A). As MIF is known to be stored in intracellular pools, being secreted only after stimulation [57, 58, 59, 60], high MIF‐expressing CATT_7_ mice, MIF^−/−^ mice, and WT mice were exposed to a model of house dust mite‐induced acute allergic airway inflammation prior to generating BMDM supernatants. The supernatants from BMDMs isolated from WT, MIF^−/−^, and CATT_7_ mice were measured by human MIF ELISA, where only CATT_7_ BMDM supernatant contained high levels of human MIF, thus serving as a source of endogenous MIF (Figure 2B).

VEGF, an important soluble factor secreted by MSCs [16, 19, 61], is a trophic factor known to play a role in wound healing [62]. After licensing MSCs with rhMIF, human VEGF (hVEGF) levels in rhMIF‐MSC CM supernatants were measured by ELISA (Figure 2C). MSCs stimulated with all concentrations of rhMIF exhibited significantly elevated levels of hVEGF in a dose‐dependent manner. MSCs licensed with 100 and 400 ng/mL rhMIF secreted the highest hVEGF protein levels, with no statistical difference between the two concentrations (Figure 2C). Thus, 100 ng/mL of rhMIF was used to license MSCs for the remainder of the study.

Alternatively, after licensing MSCs with CATT_7_‐derived human MIF, WT‐derived murine MIF, or no MIF (MIF^−/−^), VEGF protein levels in the endogenous MIF‐MSC CM were measured by ELISA (Figure 2D). MIF^−/−^ and murine MIF‐expressing WT mice did not significantly increase VEGF production by MSCs compared with cDMEM control (Figure 2D). Conversely, MSCs licensed with CATT_7_ supernatants containing human MIF displayed significantly elevated VEGF protein levels in the associated conditioned media, compared with those licensed with MIF^−/−^ or WT supernatants. This shows that human, but not murine MIF, drives enhanced VEGF production by human MSCs (Figure 2D).

### 3.3 MIF Signaling Through CXCR4, but Not CXCR2 or CD74, Leads to Enhanced VEGF Production in MSCs

MIF licensing significantly increased MSC production of VEGF (Figure 2). As previously discussed, MIF signals through canonical (CD74) and non‐canonical receptors (CXCR2 and CXCR4), are all constitutively expressed on the surface of MSCs (Figure 1). Thus, to further validate the involvement of MIF in the licensing of MSC cytoprotective ability, MSCs were exposed to an anti‐CD74 neutralising antibody (10 mg/mL) or immunoglobulin G1 (IgG1) isotype control (10 mg/mL) for 30 min before being incubated with CATT_7_ CM for 24 h. Similarly, a CXCR2 chemokine receptor inhibitor (Reparixin; 40 µM) or a CXCR4 chemokine receptor inhibitor (AMD3100) (50 µg/mL) were used to determine if MIF's non‐canonical receptors were responsible for the MIF‐MSC interaction, resulting in enhanced VEGF production into the MSC secretome.

**FIGURE 3:**
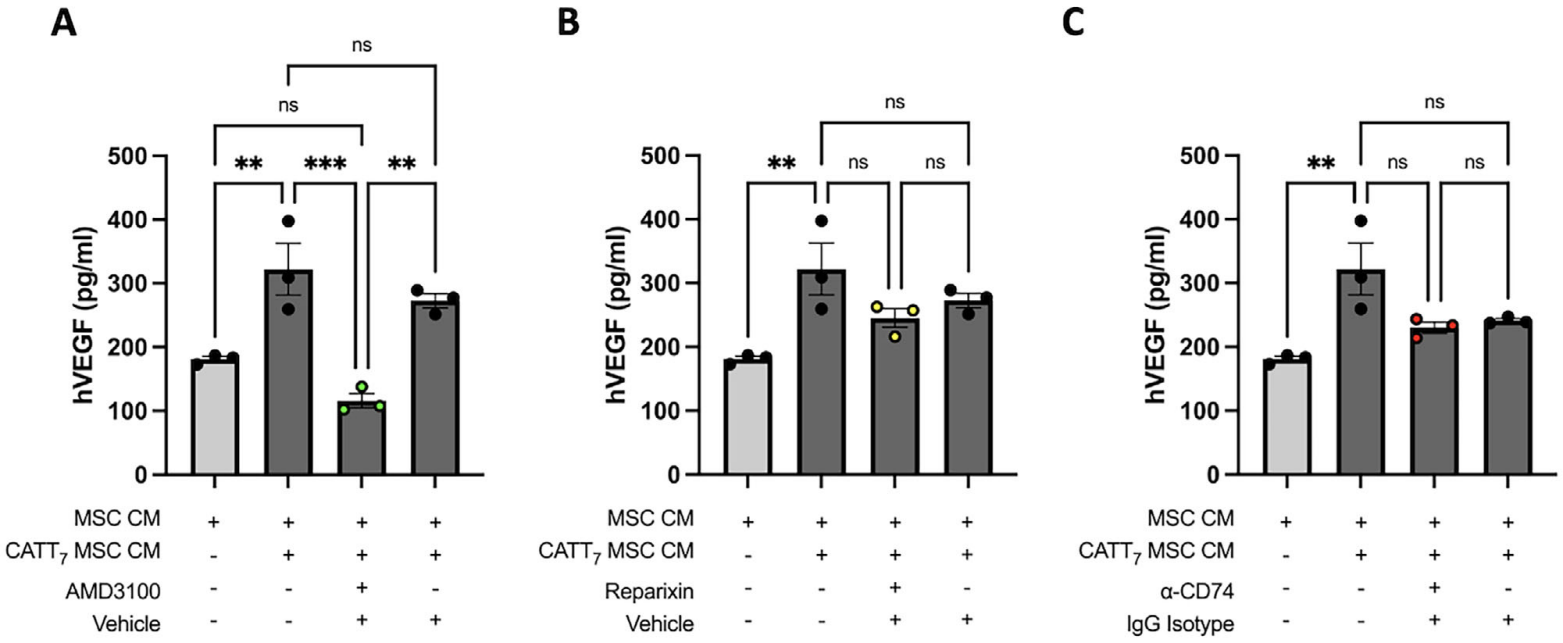
MIF signaling through CXCR4, but not CXCR2 or CD74 leads to enhanced VEGF production in MSCs. MIF MSC conditioned media was generated by licensing MSCs with endogenous human MIF secreted by BMDMs of HDM‐challenged CATT_7_ transgenic mice. To investigate the MIF receptor involved in enhanced VEGF production by MSCs, inhibitors of (A) CXCR4 (AMD3100), (B) CXCR2 (reparixin), or (C) a CD74 neutralizing antibody were used. Prior to endogenous MIF licensing, MSCs were exposed to inhibitors, vehicle control (DMSO), or IgG Isotype control for 30 min. After 24 h, media was removed and cells were washed with PBS before being replaced with serum‐free media for 48 h. VEGF production was measured by human VEGF ELISA. Data are presented as mean ± SEM and represent three independent experiments. Statistical significance was determined by one‐way ANOVA; ns = non‐significant; *p < 0.05, **p < 0.01, ***p < 0.001, ****p < 0.0001.

CATT_7_‐MSC CM had significantly increased levels of VEGF production compared with MSCs that were not licensed (Figure 3A). Interestingly, MSCs that were pre‐treated with the CXCR4 chemokine receptor inhibitor AMD3100 but not vehicle control prior to CATT_7_ CM licensing had significantly less VEGF production compared with MSCs that were licensing with CATT_7_ CM alone (Figure 3A), demonstrating that MIF licensing of MSCs was dependent on the CXCR4 receptor. No significant difference was noted between reparixin pre‐treated CATT_7_‐MSC CM and vehicle control CATT_7_‐MSC CM groups, indicating that MIF‐MSC signalling is independent of CXCR2 (Figure 3B). Lastly, MSCs pretreated with the CD74 neutralizing antibody prior to CATT_7_‐CM licensing did not have significantly different levels of VEGF compared with MSCs licensed with CATT_7_‐CM (Figure 3C). Similarly, the addition of an IgG1 isotype control prior to MSCs being licensed with CATT_7_‐CM had no significant effect on MSC production of VEGF, concluding that MIF also does not signal through the CD74 receptor on the surface of MSCs (Figure 3C). Thus, these data conclude that MIF signaling through CXCR4, but not CXCR2 or CD74 leads to enhanced VEGF production in MSCs.

### 3.4 Endogenous CATT_7_‐MIF MSC Conditioned Media Drives Bronchial Epithelial Wound Closure in a VEGF‐Dependent Manner

**FIGURE 4:**
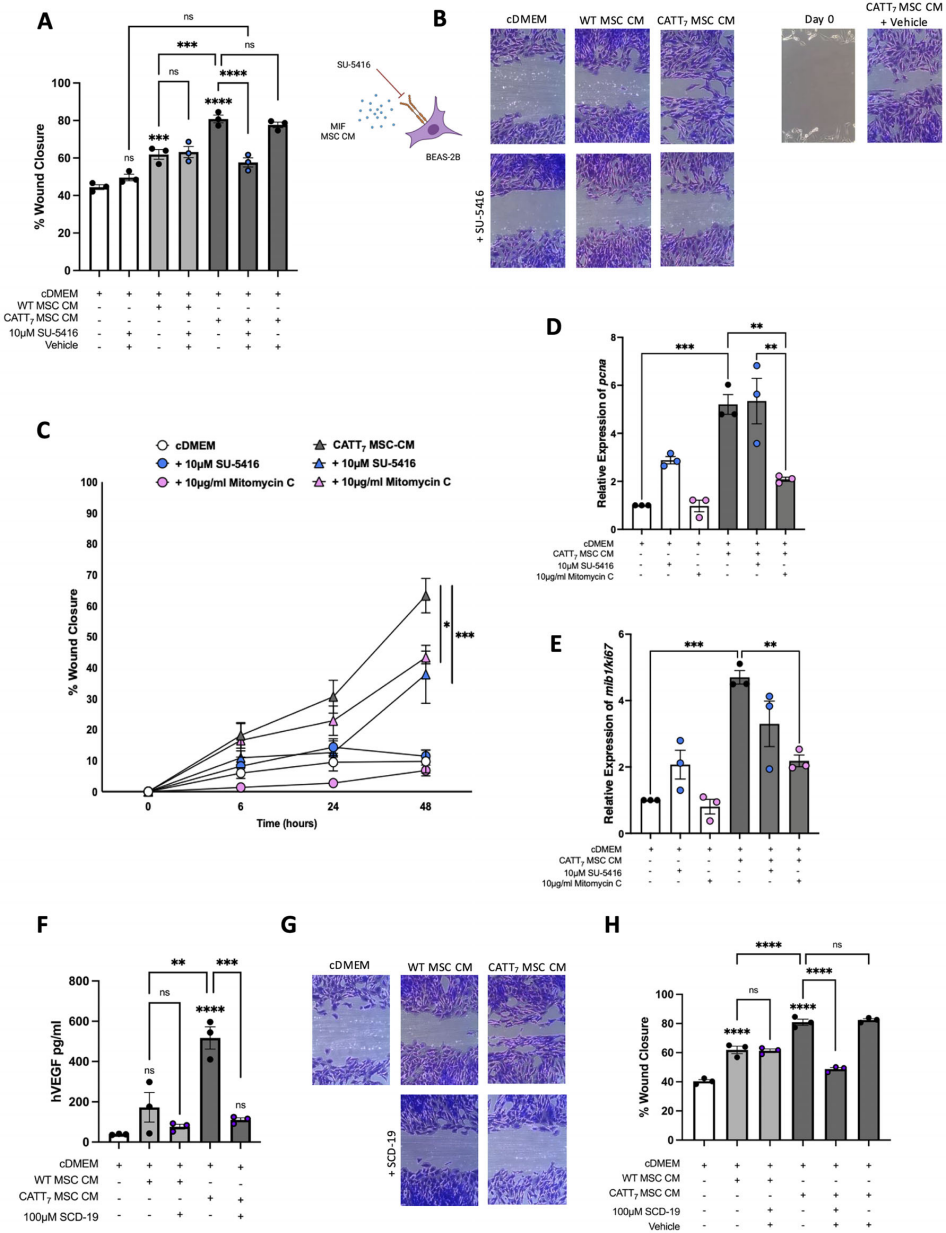
Endogenous CATT_7_‐MIF MSC conditioned media drives bronchial epithelial wound closure in a VEGF‐dependent manner. Using a human bronchial epithelial cell line (BEAS‐2B), a scratch assay was carried out to investigate the impact of MIF MSC CM in bronchial cell wound closure. A, B, A VEGFR2 inhibitor SU‐5416 (10 µM) or vehicle control (DMSO) was added to BEAS‐2B for 4 h prior to the addition of WT or CATT_7_ MIF MSC CM. C, The cell cycle inhibitor mitomycin C (10 µg/mL) was added to BEAS‐2B for 2 h before being washed off with PBS prior to the addition of cDMEM or CATT_7_ MIF MSC CM. Wound healing was quantified at 6, 24, and 48 h and cells were harvested for gene expression analysis of (D) *pcna* and (E) *ki67* at 48 h. F–H, To block MIF activity, a MIF inhibitor SCD‐19 (100 µM) or vehicle control (DMSO) was added to WT and CATT_7_ BMDM supernatants for 1 h before being added to BEAS‐2B cells. After 48 h, or until the first scratch had closed, media was removed and cells were fixed with 10% neutral buffered formalin and stained with crystal violet before being imaged using Optika imaging software on a Nikon imaging microscope. Percentage wound closure relative to day 0 baseline was calculated using Image J software. Data are presented as mean ± SEM and represent three independent experiments. Statistical significance was determined by one‐way ANOVA in (A, D–F, H) and two‐way ANOVA in (C); ns = non‐significant; *p < 0.05, **p < 0.01, ***p < 0.001, ****p < 0.0001.

In the asthmatic lung, repeated exacerbations can inflict injury on the membrane epithelium of the lung. Furthermore, the role of VEGF in wound healing is established in a variety of different conditions [62, 63, 64], such as type 1 diabetes [65] and pulmonary fibrosis [66]. We have shown that the increased levels of VEGF observed in CM from rhMIF‐licensed MSCs (Figure 2C) and endogenous CATT_7_‐MSC CM (Figure 2D) drive airway epithelial cell wound closure in human alveolar basal epithelial cells; A549s (Figure S2B,C). Using the more physiologically relevant normal human bronchial epithelial cell line (BEAS‐2B), we show that CATT_7_‐MSC CM significantly increased the percentage of wound closure compared with WT‐MSC CM and cDMEM groups (Figure 4A) (Table S1). To validate the role of VEGF in bronchial epithelial wound closure, a potent and specific VEGFR2 inhibitor, SU‐5416 was used to block the VEGF receptor on the surface of epithelial cells prior to the addition of CATT_7_‐MSC CM (Figure 4A). Blocking VEGFR2 on BEAS‐2B cells attenuated the significant enhancement of wound closure mediated by CATT_7_‐MSC CM, but not WT‐MSC CM (Figure 4A,B), illustrating the importance of human CATT_7_ MIF licensing in MSC‐derived VEGF production and BEAS‐2B wound closure, and that murine MIF does not drive wound closure in a VEGF‐dependent manner. Similarly, the application of SU‐5416 to A549s demonstrated the important role of MIF licensing on MSC's cytoprotective abilities (Figure S2B,C). BEAS‐2B cells pretreated with the vehicle control prior to the addition of CATT_7_‐MIF MSC CM maintained a significant increase in percentage wound closure mediated by CATT_7_‐MIF MSC CM. The use of a VEGFR2 inhibitor had no off‐target, non‐specific effects on the general growth of these cells, as cDMEM wells treated with SU‐5416 had no significant difference in percentage wound closure compared with cDMEM alone (Figure 4A). The increase in wound closure associated with CM from CATT_7_‐MIF licensed MSCs is illustrated in Figure 4B. Furthermore, when the VEGFR2 was blocked using SU‐5416 but not vehicle control, the inhibition of wound closure was clear (Figure 4B).

To prove that conditioned media from another cell type could not facilitate enhanced wound closure in alveolar epithelial cells, or that a positive VEGF feedback loop in A549 conditioned media was encouraging self‐renewal of these cells, conditioned media from A549 cells was used as a negative control. A549 conditioned media did not significantly increase the percentage of wound closure in A549 epithelial cells (Figure S2B,C).

We have elucidated that human, but not murine, MIF drives VEGF production from MSCs (Figure 4F) and thus facilitates a significant increase in wound closure (Figure 4A,B). We performed experiments to determine the effect of SU‐5416 on the proliferation of BEAS‐2B cells. In wound closure experiments, the addition of SU‐5416 had no significant effect on BEAS‐2B cells cultured in cDMEM (Figure 4C); however, SU‐5416 significantly reduced wound closure mediated by CATT_7_ MSC‐CM (Figure 4C). We also used mitomycin C, a cell cycle antagonist to inhibit cell proliferation. We show that the addition of mitomycin C significantly reduced wound closure mediated by CATT_7_ MSC‐CM (Figure 4C). We also examined the expression of two genes associated with proliferation; *pcna* and *ki67*. CATT_7_ MSC‐CM significantly increased the expression of *pcna* and *ki67* in BEAS‐2B cells (Figure 4D,E). While SU‐5416 had no significant effect on BEAS‐2B cells exposed to CATT_7_ MSC‐CM, the addition of mitomycin C significantly decreased the expression of *pcna* and *ki67* (Figure 4D,E). Together, these findings support a role for both migration and proliferation in the enhancement of wound closure mediated by CATT_7_ MSC‐CM.

To fully elucidate this MIF‐associated effect on MSC CM increase in wound closure, an MIF inhibitor SCD‐19 was used to block MIF's biological activity prior to MSC licensing. When endogenous CATT_7_ MIF supernatants were incubated with 100 µM of SCD‐19 for 1 h prior to MSC licensing, MIF inhibition significantly decreased MSC‐mediated VEGF production compared with cDMEM controls (Figure 4F). Importantly, SCD‐19 did not affect WT‐derived MIF supernatants, as no significant difference in VEGF production was noted.

SCD‐19, but not vehicle control, effectively decreased the capacity for CATT_7_‐MSC CM to enhance wound closure in bronchial epithelial cells (Figure 4G,H). These data conclude that high levels of human MIF from CATT_7_ BMDM‐derived supernatants can license MSCs to produce increased levels of VEGF (Figures 2D and 4F), with increased efficacy than those licensed with recombinant MIF (Figure 2C). Following this narrative, conditioned media generated from endogenous human MIF‐licensed MSCs can significantly increase wound closure in BEAS‐2B cells in a VEGF‐dependent manner (Figure 4A,B), illustrating MIF's specific role through utilising a potent MIF antagonist SCD‐19 (Figure 4G,H).

### 3.5 VEGF Produced by MIF‐Licensed MSCs Reduces HDM‐Induced Goblet Cell Hyperplasia

**FIGURE 5:**
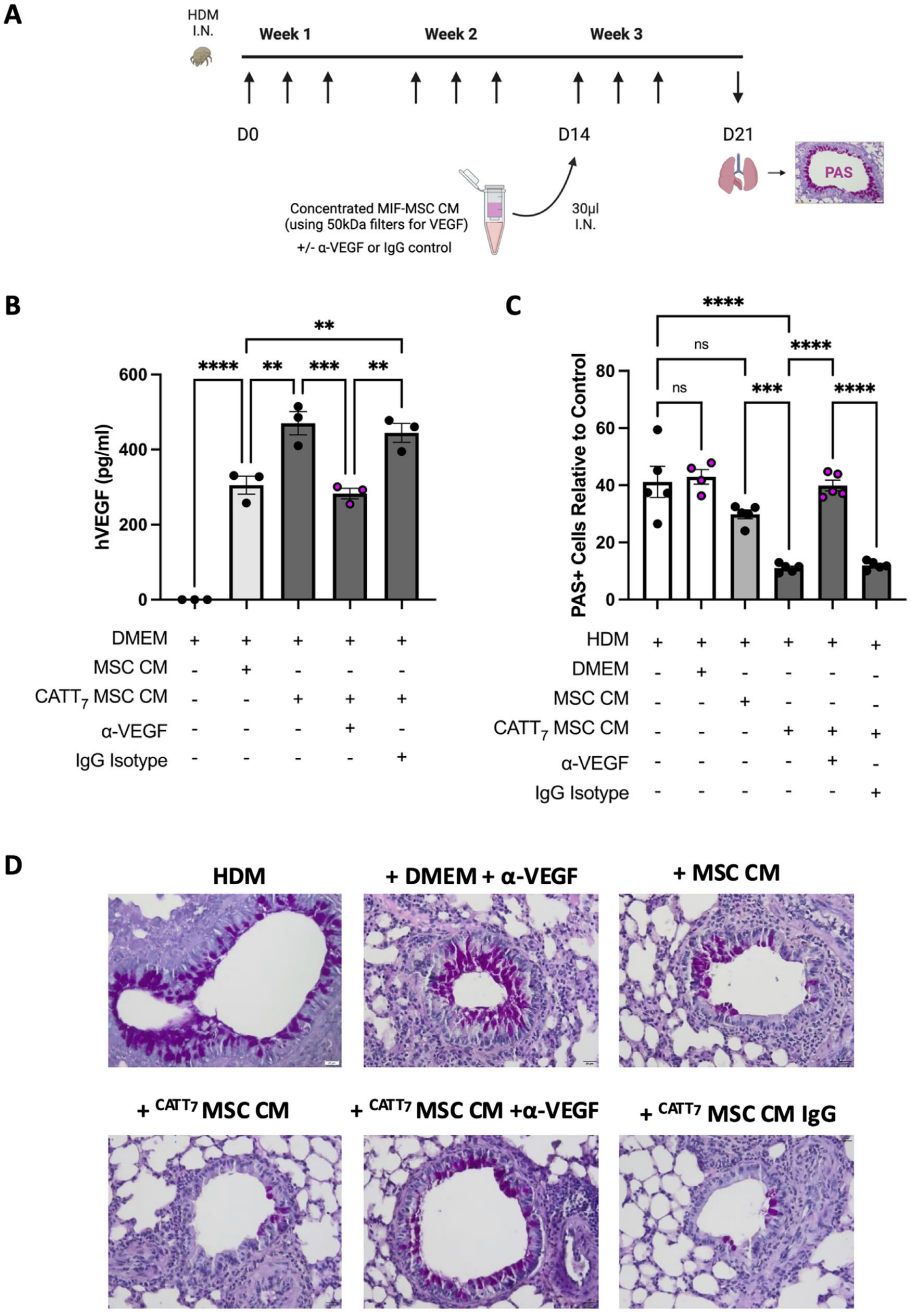
VEGF produced by MIF‐licensed MSCs reduces HDM‐induced goblet cell hyperplasia. A, CATT_7_ mice were challenged with 25 µg of HDM intranasally I.N. three times a week for 3 weeks. MIF MSC CM containing VEGF was concentrated using 50 kDa Amicon filters. A, VEGF‐neutralizing antibody (8 ng/mL) or IgG isotype control was added before intranasal administration on day 14. B, VEGF levels were measured by VEGF ELISA. C, D, Lungs were harvested on day 21 for histological analysis by staining goblet cells with periodic acid Schiff (PAS). Slides were imaged and the number of PAS‐positive cells (magenta) relative to the control were quantified using ImageJ software. *N* = 4–5 mice per group. Data are presented as mean ± SEM. Statistical significance was determined by one‐way ANOVA; ns = non‐significant, **p < 0.01, ***p < 0.001, ****p < 0.0001.

VEGF, specifically through VEGFR2 (KDR) signalling, has previously been shown to protect against goblet cell metaplasia during processes of goblet cell renewal [67]. Thus, we set out to investigate the therapeutic capacity of VEGF present in CATT_7_‐MIF MSC CM in an HDM model of allergic airway inflammation. To elucidate the specific role of MSC‐derived VEGF in vivo, an anti‐human VEGF neutralising antibody or IgG isotype control was used (Figure 5A).

After MSC CM and CATT_7_‐MIF MSC CM were generated as previously described (Figure 2A), supernatants were concentrated using ultracentrifugal filters with a molecular cut‐off of 50 kDa (VEGF molecular weight: 45 kDa) to allow for intranasal administration on day 14 of the HDM model (Figure 5A). To neutralise MSC‐derived VEGF present in conditioned media, a bevacizumab biosimilar anti‐VEGF monoclonal antibody or IgG isotype control was added to the supernatant at 8 ng/mL prior to I.N. administration on day 14.

To ensure the efficacy of the VEGF neutralising antibody, VEGF protein in the MSC CM was measured using a human VEGF ELISA. As expected, CATT_7_ MSC CM contained significantly elevated levels of VEGF compared with that present in conditioned media from unlicensed MSCs (Figure 5B). CATT_7_ MSC CM + α‐VEGF had a significantly decreased concentration of VEGF present, compared with both CATT_7_ MSC CM, and CATT_7_ MSC CM + IgG isotype control (Figure 5B).

To investigate the therapeutic role of CATT_7_ MSC CM in a model of HDM‐induced allergic asthma, lungs were harvested on day 21 and processed for histological analysis, tissue sections were stained with PAS to visualise and measure levels of airway goblet cell hyperplasia. CATT_7_ MSC CM, but not MSC CM significantly reduced HDM‐induced goblet cell hyperplasia (Figure 5C,D), reiterating the importance of human MIF in licensing MSC's cytoprotective efficacy. However, administration of CATT_7_ MSC CM treated with α‐VEGF neutralising antibody, but not an IgG control antibody could no longer significantly decrease the number of PAS‐positive cells in HDM‐challenged mice, demonstrating the role of VEGF in epithelial protection against goblet cell hyperplasia. This study demonstrates that MIF‐licensing significantly increases VEGF production by MSCs which is responsible for the enhanced cytoprotective effects of CATT_7_‐licensed MSC CM in airway epithelial cells in vitro and in a clinically relevant pre‐clinical model of HDM‐induced allergic airway inflammation.

## 4 Discussion

This study set out to investigate the cytoprotective mechanisms associated with the MIF‐licensed MSC secretome in the context of epithelial injury in vitro and in vivo. Human BM‐MSCs express the canonical (CD74) and non‐canonical (CXCR2 and CXCR4) MIF receptors allowing MSCs to respond to MIF present in the microenvironment. Previously, we have demonstrated that MIF can promote the expansion and immunosuppressive function of human BM‐MSCs in vitro and significantly increase the retention of human BM‐MSCs in vivo in an HDM model of allergic asthma [42]. MIF signals through different receptors depending on the function. We have previously shown that CD74 is required for MIF‐licensed MSC immunomodulation and to significantly increase the retention of human BM‐MSCs in vivo in an HDM model of allergic asthma [42]. Differentially, CXCR4 has been shown to mediate MSC chemotaxis to MIF [68]. Here we show that MIF‐licensing significantly enhances MSC secretion of VEGF in a CXCR4‐dependent manner. In addition to rhMIF, endogenous human MIF produced by CATT_7_ BMDMs also had the capacity to license MSCs leading to significantly increased MSC‐derived VEGF secretion. Importantly, the use of WT BMDM CM to license MSCs did not have the same effect. This novel finding indicates that human, but not murine, MIF boosts the production of MSC‐derived VEGF. This supports the idea that the communication between exogenously administered MSCs and macrophages plays an important role in dictating MSC therapeutic effects.

MIF has previously been shown to upregulate VEGF production in the conditioned media of synovial fluid mononuclear cells [69] and endometrial stromal cells [70] promoting new blood vessel formation. Functionally, CATT_7_‐MIF licensed MSC CM exerted superior wound healing capacity in normal bronchial epithelial cells (BEAS‐2Bs) compared with naive MSC CM or WT‐licensed MSC CM. MSC CM has been previously shown to promote wound healing in airway epithelial cells via growth factors including HGF and KGF [23, 71].

Our group and others have highlighted the importance of licensing MSCs to enhance their therapeutic efficacy [42, 72, 73], however, less is understood about the influence of licensing approaches on MSC cytoprotective functions. Licensing approaches using IFNγ and hypoxia [74], or IFNγ and TNFα [75] have previously been used to enhance the cytoprotective/wound healing properties of MSC CM. VEGF production from MSCs has been shown to be increased by licensing MSCs with hypoxia [76, 77, 78, 79, 80], fibroblast growth factor‐2 [81], TGF‐α [82], IL‐1β [83], and LPS [84]. However, we are the first to demonstrate enhanced VEGF production from MSCs licensed with human MIF, a clinically relevant proinflammatory cytokine in a range of inflammatory diseases including asthma.

Blockade of MIF using the small molecule inhibitor, demonstrated the specificity of MIF in licensing MSCs to produce significantly increased levels of VEGF. This provides a novel mechanistic insight into how MIF can license MSCs and enhance their cytoprotective function, specifically their ability to provide protection against a dysregulated airway epithelial barrier and thus, also prevent the development of airway goblet cell hyperplasia after allergen challenge in vivo. The limitation of our study is that although we show a role for MSC‐CM‐derived VEGF, we have not identified the mechanism of how this MSC‐CM‐derived VEGF contributes to the repair of HDM‐induced epithelial damage in mice. Moreover, our findings of effects mediated by human VEGF on mouse epithelial cells in vivo add further complexity. However, human VEGF has been shown to act on mouse epithelial cells both in vitro and in vivo [85, 86].

VEGF has historically played a central role in epithelial repair and the maintenance of epithelial barrier integrity, where VEGF‐deficient mice had increased levels of bronchial and alveolar apoptosis [87]. VEGF enhanced wound healing, survival, and proliferation of airway epithelial cells [88, 89]. Furthermore, VEGF overexpression reduced bleomycin‐induced cell death in a model of idiopathic pulmonary fibrosis [66]. Interestingly, VEGF‐A and its receptor VEGFR2 have been shown to have a protective role in the defence against mucous cell metaplasia, commonly documented in asthma and cystic fibrosis [67]. In asthma, if VEGF‐A levels are decreased, the transcription factor Sox9 is upregulated, driving the club to goblet cell differentiation, exacerbating disease [67]. These studies, along with the data presented throughout this manuscript depict the protective role of VEGF in the repair and regulation of the airway epithelial barrier, decreasing goblet cell hyperplasia after the HDM challenge in vivo.

In the lung, there has been evidence of MSC‐derived VEGF having a protective role in acute lung injury [90, 91]; however, the effect of MSC‐secreted VEGF in allergic asthma is unknown. Thus, this manuscript is the first to demonstrate the impact of MSC‐VEGF on epithelial cells in vitro, and in vivo in a clinically relevant model of HDM‐induced acute allergic asthma.

## Author Contributions

Hazel Dunbar performed research, analysed the data, designed the study, and wrote the manuscript. Ian J. Hawthorne performed research, analysed the data, and designed the study. Courteney Tunstead performed research and analysed the data. Molly Dunlop and Evelina Volkova performed research. Daniel J. Weiss and Claudia C. dos Santos contributed to the study design and data analysis. Seamas C. Donnelly and Michelle E. Armstrong provided reagents and contributed to the study design and data analysis. Karen English designed and supervised the study and wrote the manuscript. All authors approved the final manuscript.

## Conflicts of Interest

The authors declare no conflicts of interest.

### Peer review

The peer review history for this article is available at https://publons.com/publon/10.1002/eji.202451205.

## Supporting information



Supporting Information

## Data Availability

The data that support the findings of this study are available from the corresponding author upon reasonable request.
