# Supplementary material for: The VEGF‐Mediated Cytoprotective Ability of MIF‐Licensed Mesenchymal Stromal Cells in House Dust Mite‐Induced Epithelial Damage
Source: Eur J Immunol. 2024 Nov 6;55(1):e202451205. doi: 10.1002/eji.202451205 (PMC11739667; doi:10.1002/eji.202451205)
Supplement: Supplementary file 1 — Supporting Information [file EJI-55-e202451205-s001.pdf]

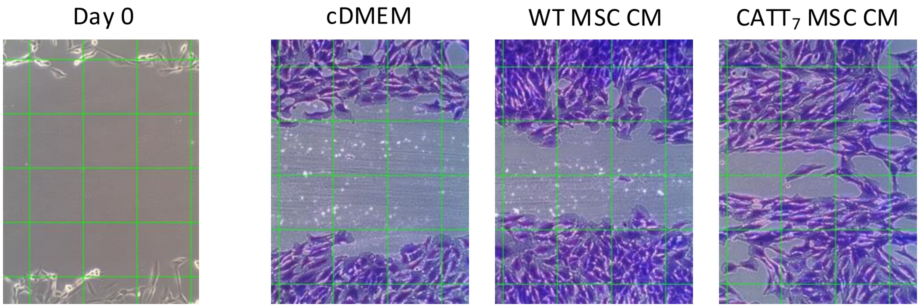

Supplementary Figure 1. Measurement of percentage wound closure using Image J grid tool. Using a human bronchial epithelial cell line (BEAS-2B), a scratch assay was carried out to investigate the impact of WT or MIF MSC CM compared to cDMEM in bronchial cell wound closure. Percentage wound closure relative to day 0 baseline was calculated using Image J software. Representative images of BEAS-2B wound closure after crystal violet stain are shown.

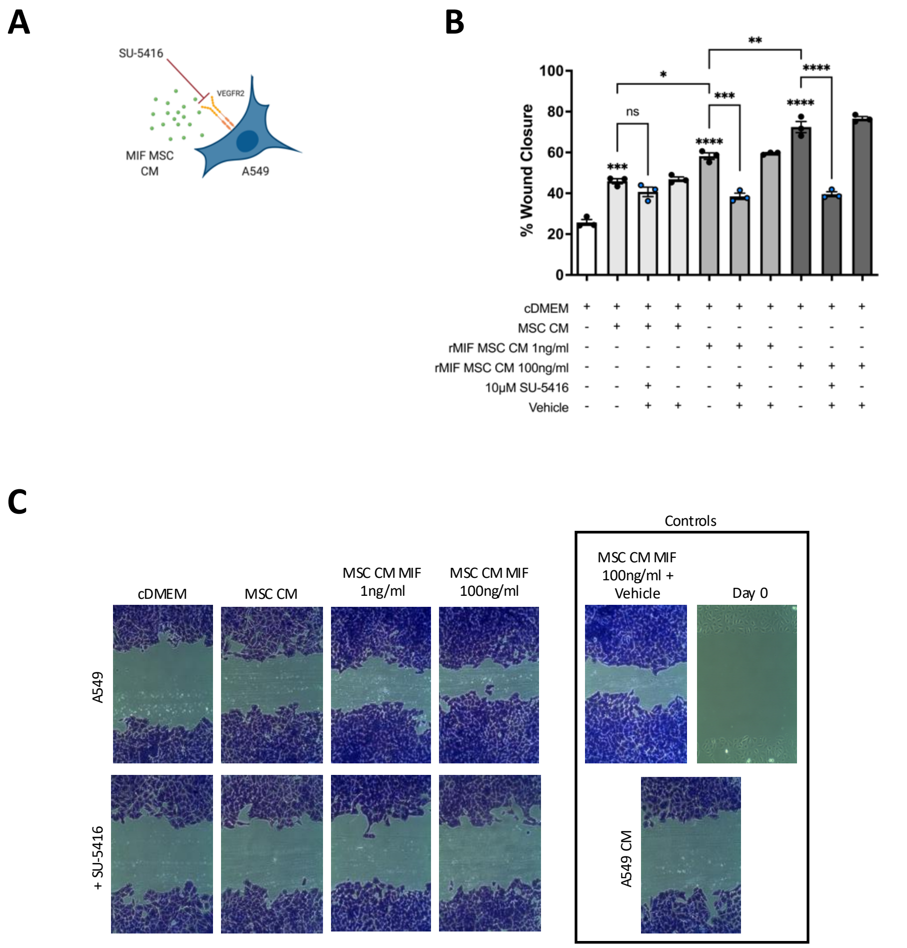

Supplementary Figure 2. rhMIF licensing of MSCs significantly enhances wound healing in human alveolar basal epithelial cells in a VEGF dependent manner. A Human VEGF in recombinant MIF MSC CM binds to A549 epithelial cells through the VEGFR2 receptor. SU-5416, a VEGFR2 inhibitor, blocks this interaction. B Percentage wound closure relative to the control in alveolar basal epithelial cells (A549s) after stimulation with rhMIF MSC CM. C Images of A549 wound closure after crystal violet stain. Data are presented as mean ± SEM; images are representative of 3 independent experiments. ns=non significant, *p<0.05, **p<0.01,***p<0.001, ****p<0.0001.

Measurement	Day 0	cDMEM	WT MSC CM	$CATT_7$ MSC CM
1.	832	465	315	220
2.	838	587	343	154
3.	852	452	264	154
4.	896	480	328	141
Avg	854.5	496	312.5	167.25
% Open	100	58.05	36.57	19.57
% Closed	-	41.95	63.43	80.43

Supplementary Table 1. Calculation of percentage wound closure in bronchial epithelial cells. Percentage wound closure relative to day 0 baseline was calculated using Image J software.

Gene	Supplier	Species	Forward Sequence	Reverse Sequence
hprt	Sigma™	Human	5’ATAAGCCAGACTTTGTTGG	5’ATAGGACTCCAGATGTTTCC
pcna	Sigma™	Human	5’CTGTGTAGTAAAGATGCCTTC	5’TCTCTATGGTAACAGCTTCC
Mib1/ki67	Sigma™	Human	5’AAAGGTAACAGAAATCCAGG	5’CCAACTCTGTAAAGGTTCTTAG

Supplementary Table 2. Primer Sequences
